# Considerations in the Care of Athletes With Type 1 Diabetes Mellitus

**DOI:** 10.7759/cureus.22447

**Published:** 2022-02-21

**Authors:** George Pujalte, Hebah M Alhumaidi, Kenneth Patrick L Ligaray, Rock P Vomer, Krishna Israni, Andre A Abadin, Shon E Meek

**Affiliations:** 1 Division of Sports Medicine, Mayo Clinic, Jacksonville, USA; 2 Division of Endocrinology, Mayo Clinic, Jacksonville, USA; 3 Endocrinology, University of Wisconsin, Madison, USA; 4 Family Medicine, Eastern Virginia Medical School, Norfolk, USA; 5 Department of Family Medicine, Mayo Clinic, Jacksonville, USA

**Keywords:** insulin, hyperglycemia, hypoglycemia, glucose, athlete, diabetes

## Abstract

Type 1 diabetes mellitus is an autoimmune disease caused by affected individuals’ autoimmune response to their own pancreatic beta-cell. It affects millions of people worldwide. Exercise has numerous health and social benefits for patients with type 1 diabetes mellitus; however, careful management of blood glucose is crucial to minimize the risk of hypoglycemia and hyperglycemia. Anaerobic and aerobic exercises cause different glycemic responses during and after exercise, each of which will affect athletes’ ability to reach their target blood glucose ranges. The optimization of the patient’s macronutrient consumption, especially carbohydrates, the dosage of basal and short-acting insulin, and the frequent monitoring of blood glucose, will enable athletes to perform at peak levels while reducing their risk of dysglycemia. Despite best efforts, hypoglycemia can occur. Recognition of symptoms and rapid treatment with either fast-acting carbohydrates or glucagon is important. Continuous glucose monitoring devices have become more widely used in preventing hypoglycemia.

## Introduction and background

Type 1 diabetes mellitus (T1DM) is an autoimmune disease characterized by insulin deficiency due to antibody-mediated destruction of the β cells of the pancreas [1]. Many patients with T1DM depend on exogenous insulin for survival. An estimated 30.3 million people of all ages, or 9.4% of the US population, were reported to have diabetes mellitus (DM) in 2015. Among this total, 193,000 children and adolescents younger than age 20 years were diagnosed with DM. This age range is of interest, as most athletes are part of this population [2-3]. About 5% of people with DM are estimated to have T1DM [4].

The survival of patients with T1DM has improved considerably due to several advances in insulin therapy and delivery, as well as improved glucose monitoring methods [5]. Patients with T1DM are capable of competing in high-performance sports and participating in extreme fitness challenges [6-7]. Athletes have become Olympic gold medalists and professional players after being diagnosed with T1DM [8]. Many young patients with T1DM are encouraged by these exceptional athletes to participate in sports and extensive exercise regimens.

Exercise has numerous health and social benefits and should be recommended to all patients with T1DM. However, it is challenging to manage blood glucose during exercise in patients with T1DM due to the increased risk of exercise-induced hypoglycemia or hyperglycemia. Managing blood glucose in athletes with T1DM requires an understanding of the physiology of glucose homeostasis during and after exercise by the physician and patient. The aim of this review is to describe the physiology of glucose metabolism in response to exercise, the benefits of exercise in T1DM, recommendations for glycemic control, and blood glucose monitoring during and after exercise in athletes with T1DM.

## Review

Glycemic response to exercise

Exercise is largely divided into aerobic and anaerobic based on the energy-producing system used. Aerobic exercise, including walking, running, dancing, hiking, and cycling, involves continuous and rhythmic involvement of large muscle groups and is largely dependent on inhaled oxygen in conjunction with glucose and fat as energy sources [4]. The American College of Sports Medicine defines anaerobic exercise as intense physical activity for a very short duration, fueled by energy sources within the contracting muscles [5]. Anaerobic exercise consists of resistance exercises such as resistance training with weights or bands and high-intensity interval training (HIIT). HIIT involves brief, high-intensity, anaerobic exercise for six seconds to four minutes separated by intervals of low-intensity aerobic exercise, or rest, ranging from 10 seconds to five minutes [6].

Several factors affect glycemic control during exercise in patients with T1DM, including the insulin delivery method, amount of insulin in the circulation, site of insulin injection, blood glucose level before exercise, composition of the meal before exercise, and duration and intensity of exercise [7].

During aerobic exercise, muscle glucose uptake increases up to five-fold through insulin-independent mechanisms. In nondiabetic individuals, insulin secretion decreases and glucagon secretion increases to promote glycogenolysis and prevent hypoglycemia during aerobic exercise. After exercise, glucose uptake remains elevated for up to 48 hours to restore glycogen to the normal level [8]. Mild to moderate aerobic exercise has been associated with post-exercise hypoglycemia in T1DM, as the circulating insulin level does not drop at the start of exercise [9]. Late-onset hypoglycemia more commonly occurs after morning exercise as compared to afternoon exercise in patients with T1DM [10].

When compared to aerobic exercise, resistance exercise is associated with a less initial decline in blood glucose during exercise, but a more prolonged reduction in blood glucose during the recovery period [11]. Post-exercise hyperglycemia can be seen after high-intensity aerobic and anaerobic exercises, mostly mediated by counter-regulatory hormones [12]. Stable post-exercise glucose levels are achieved with a mix of aerobic and anaerobic exercises [13]. For example, performing a 10-second sprint immediately before moderate-intensity exercise prevents hypoglycemia during the early recovery period in patients with T1DM [14]. However, findings have been mixed in ensuing trials looking into whether or not vigorous-intensity interval exercise protects against hypoglycemia [15]. Often, glucose variability rates were not significantly different among exercise arms, and in some studies, hypoglycemic events were more frequent after the highest intensity interval sessions, relative to other experimental and control conditions [16-17]. Collectively, trials show that vigorous-intensity interval training for stabilizing blood glucose following moderate-intensity endurance exercise or preventing exercise-related hypoglycemia in individuals with T1DM may not be a practical approach [18].

Benefits of exercise in patients with T1DM

Physical activity has been shown to improve physical fitness and muscular strength in patients with T1DM [19]. Moderate to high-intensity resistance exercise has been associated with an improved lipid profile and a decrease in insulin dosage [20]. Improved insulin sensitivity, blood pressure, lipid profiles, cardiovascular health, mental health, and bone mineral density have also been seen with resistance exercise [21]. Aerobic exercise has been found to improve the lipid profile in men with T1DM, with the greatest benefit seen in already physically active men with low baseline high and low-density lipoprotein levels [22]. Furthermore, aerobic exercise has been found to reduce visceral fat mass and body weight; improve insulin sensitivity, blood pressure, and glucose control; and reduce cardiovascular risk [23]. The effect of exercise on glycated hemoglobin A1c varies. Aerobic exercise has been found to reduce hemoglobin A1c in some, but not all studies, while anaerobic exercise has not been shown to produce change at all [24-26]. The American Diabetes Association recommends physical activity to all patients with T1DM. The current recommendation is for all adults with T1DM to engage in at least 150 minutes of moderate-to-vigorous-intensity physical activity performed over at least three days weekly. However, evidence for the optimal exercise type, intensity, and duration for improved glycemic control is lacking [27].

Glycemic targets

**Figure 1:**
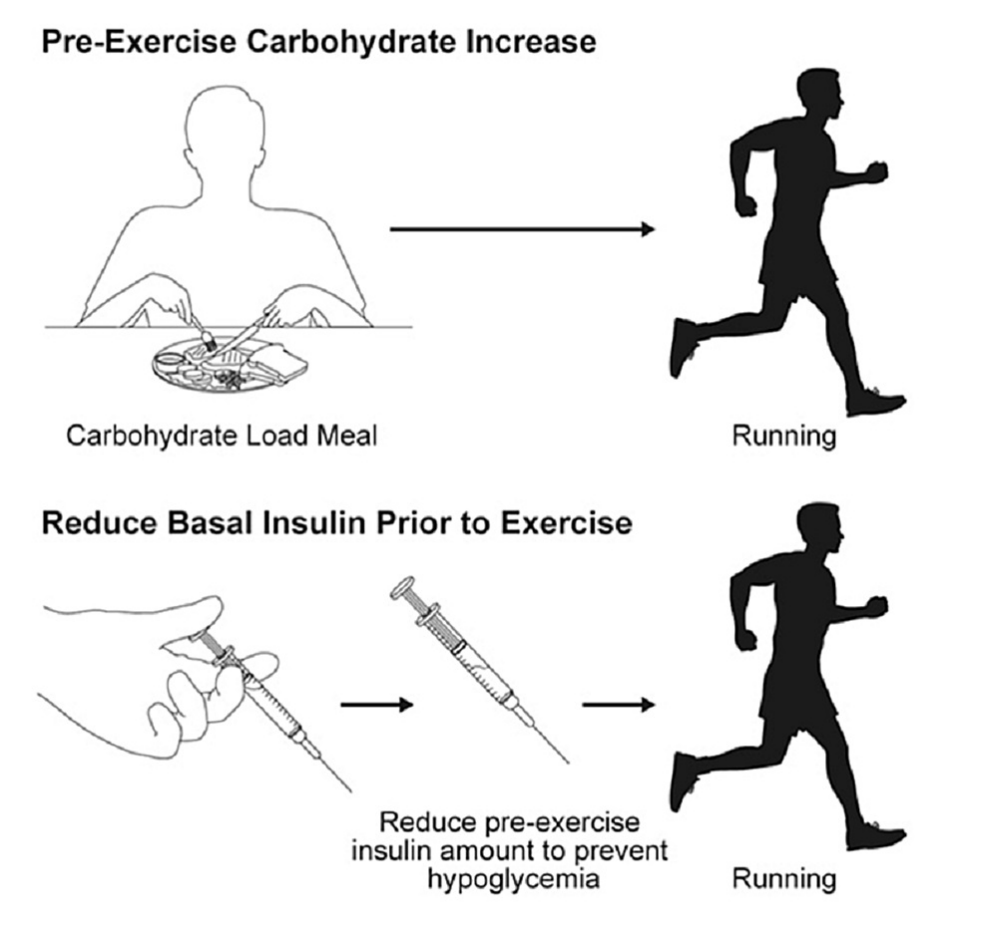
Pre-exercise carbohydrate meal and insulin reduction If pre-exercise blood glucose is between 90 and 149 mg/dL, consumption of 0.5 to 1.0 g/kg/h of carbohydrates and reducing basal insulin at the onset of exercise is recommended.

A diagnosis of T1DM should not preclude patients from performing at any level of activity, including competitive professional sports. As mentioned above, exercise induces dysglycemia in patients with T1DM, and many patients do not exercise for fear of hypoglycemia. Different approaches have been applied in the past to prevent or limit hypoglycemia, including increasing carbohydrate intake before exercise, reducing basal insulin, reducing bolus insulin for the meal before exercise, and interrupting basal insulin for patients on continuous subcutaneous insulin infusion (CSII) [24-26]. Athletes with T1DM should always check their blood glucose before exercise, looking for a target level of between 120 and 180 mg/dL [28]. If pre-exercise blood glucose is between 90 and 149 mg/dL, consumption of 0.5 to 1.0 g/kg/h of carbohydrates at the onset of exercise is recommended (Figure 1).

In patients with pre-exercise blood glucose between 150 and 249 mg/dL, consumption of extra carbohydrates is only recommended after the blood glucose drops to 150 mg/dL. Exercise should only be started after ingestion of 15 g to 30 g of carbohydrates if pre-exercise blood glucose is lower than 90 mg/dL. Testing for ketones is recommended if blood glucose is higher than 250 mg/dL [29-32]. Patients with T1DM should not exercise if their blood glucose is between 250 and 349 mg/dL and ketones are detected or if their blood glucose is 350 mg/dL or higher, even in the absence of ketones. If the blood glucose level is between 250 and 349 mg/dL and no ketones are present, mild to moderate-intensity exercise can be started, as blood glucose is expected to decrease with this type of exercise. Intense exercise should be delayed until after blood glucose is below 250 mg/dL to prevent worsening hyperglycemia. Use of insulin to correct hyperglycemia may be needed before exercise in patients with blood glucose higher than 350 mg/dL [29-32].

Nutritional recommendations for peak athletic performance

Diabetic athletes should meet certain nutritional demands to perform at peak levels. Nutritional recommendations for a diabetic athlete are similar to those of the general population. Additional calories and fluids may be required for athletes with T1DM, depending on exercise intensity, total energy expenditure, type of exercise, duration of exercise, sex, and environmental circumstances. A joint position statement by the Academy of Nutrition and Dietetics, Dieticians of Canada, and the American College of Sports Medicine recommends the following general energy requirements for competitive athletes [33]:

1. Carbohydrate consumption of 3 to 10 g/kg/d (up to 12 g/kg/d for extreme and prolonged activities).

2. Protein consumption of 1.2 to 2.0 g/kg/d for endurance and strength-trained athletes. This recommendation can generally be met through diet alone, without the use of dietary supplements.

3. Fat consumption of 20% to 35% of total energy intake. Consuming 20% or less energy from fat intake does not benefit performance.

Carbohydrates play a major role in performance and training for several reasons. First, carbohydrate consumption is important for maintaining euglycemia during and after exercise and replenishing glycogen storage. Second, carbohydrates provide the main fuel for the brain and central nervous system. Third, carbohydrates are used in both anaerobic and oxidative pathways and can support muscle exercise in various activities. It was found that depletion of glycogen storage is associated with fatigue, reduced work rate, impaired skill and concentration, and increased perception of effort. Protein aids in the synthesis of muscle and improves structural changes in other tissues such as tendons and bones. Fat is an important energy source, and it aids in the absorption of fat-soluble vitamins. Fat is an important element of cell membranes and is essential for steroid hormone synthesis, which can impact athletic performance [34].

Insulin adjustments

Patients with T1DM are either managed by a multiple daily injection (MDI) regimen or CSII. In the MDI regimen, long-acting insulin is given once or twice daily to provide basal control, and short-acting insulin is given before meals as bolus coverage for carbohydrates consumed. In CSII, an insulin pump is used to deliver continuous subcutaneous insulin as a basal coverage; bolus insulin is also given for matching the amount of carbohydrates before each meal. With the use of an insulin pump, rapid adjustments of insulin infusion can be made to meet the glycemic changes induced by exercise. It was found that among patients with T1DM performing moderate to high-intensity aerobic exercise regularly, the use of CSII limited post-exercise hyperglycemia compared with MDI therapy without increasing the risk for post-exercise late-onset hypoglycemia [35].

**Figure 2:**
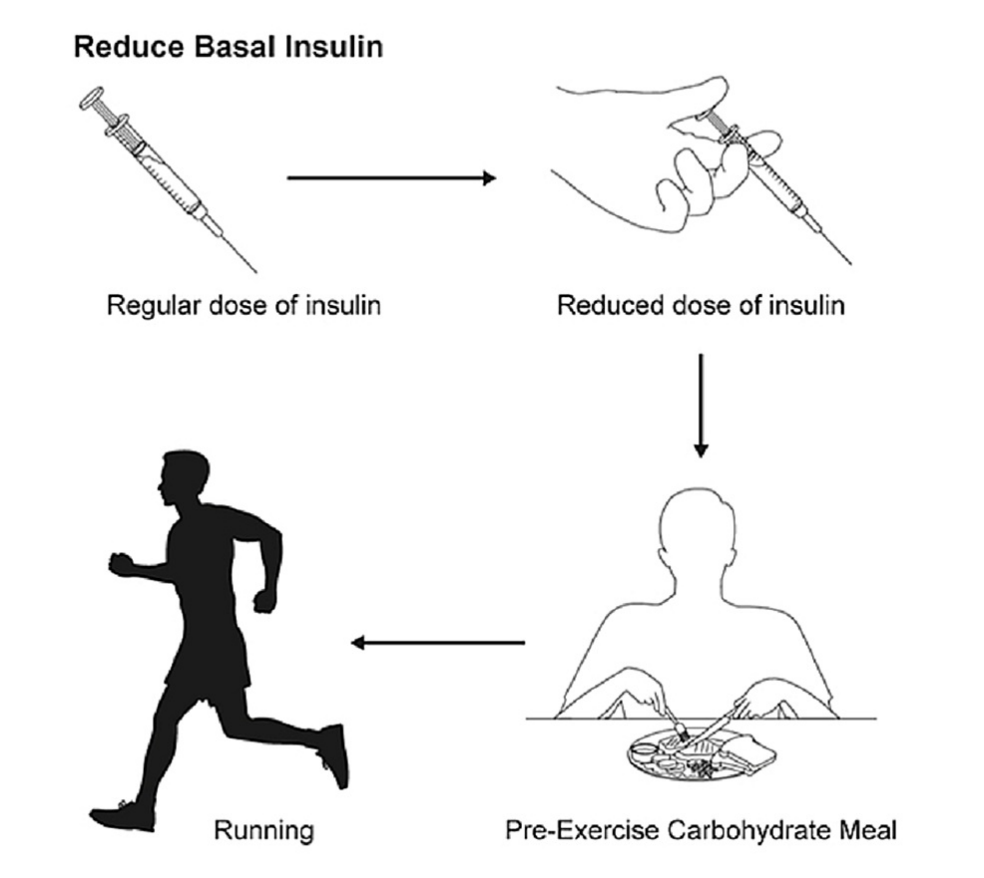
Insulin adjustment prior to exercise For patients using an MDI regimen, adjustment of meal bolus insulin should be considered for exercise performed within three hours of meal ingestion to prevent hypoglycemia. Adjustments should be based on exercise intensity. MDI: multiple daily injection

For patients using an MDI regimen, adjustment of meal bolus insulin should be considered for exercise performed within 3 hours of meal ingestion to prevent hypoglycemia (Figure 2).

For low exercise intensity (25% maximal oxygen uptake (Vo2 max)) performed for 30 minutes, a 25% reduction in pre-meal rapid insulin dose is recommended. For low exercise intensity performed for 60 minutes, and for moderate exercise intensity (50% Vo2 max) performed for 30 minutes, a 50% reduction in pre-meal rapid insulin dose is recommended. A 75% reduction in pre-meal rapid insulin dose should be used before high exercise intensity (75% Vo2 max) is performed for 30 minutes [36]. As the athlete becomes fitter, further insulin dose adjustments of 10% to 30% may be needed [37]. The abdomen is the preferred site of injection, as it has a more predictable absorption time [38]. Reducing the basal insulin dose by 20% may also be recommended before a planned tournament or excessive exercise to prevent hypoglycemia [39].

**Figure 3:**
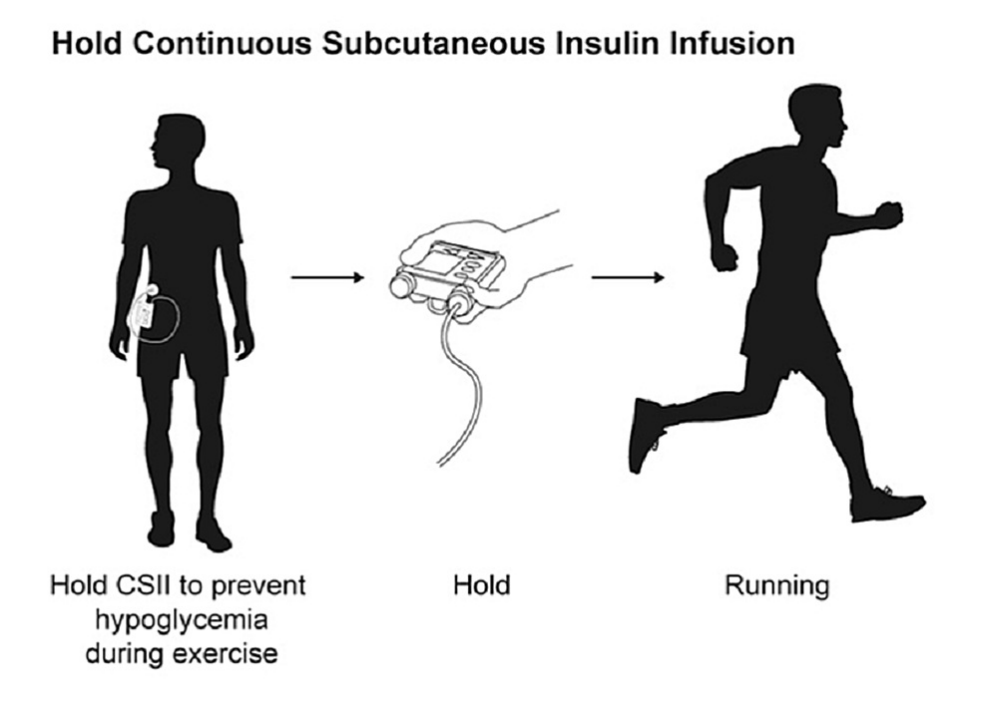
Continuous subcutaneous insulin infusion management Pump suspension should be done 60 minutes before exercise to allow time for the circulating insulin to reduce.

For patients on a CSII regimen, different strategies can be used to prevent exercise-induced dysglycemia depending on the intensity of exercise and timing in relation to meals. One strategy involves basal insulin reduction before moderate to high-intensity exercise [40]. A recent study showed that a 50% to 80% basal rate reduction set 90 minutes before exercise improved glucose control and decreased risk of hypoglycemia compared to pump suspension at exercise onset, without compromising glucose control in the post-exercise period [41]. Another approach is recommended for exercise in the post-prandial state (ie, performed within three hours after a meal), which involves reduction of bolus insulin given for the meal before exercise [42]. The proposed amount of prandial insulin reduction is similar to that of patients using an MDI regimen. Other studies recommend suspending the insulin pump before exercise [41-42]. Ideally, pump suspension should be done 60 minutes before exercise to allow time for the circulating insulin to reduce (Figure 3).

It should be noted that suspending the pump for more than 90 minutes is associated with an increased risk of hyperglycemia [42]. For contact sports or activities that involve collisions, removal of the pump 30 minutes before the activity is recommended. For exercises that last longer than one hour, small boluses of short-acting insulin given during exercise are necessary to prevent hyperglycemia [1].

Post-exercise hyperglycemia is less recognized and often goes untreated. The recommendations for post-exercise hyperglycemia treatment are conflicting. Previously, it was noted that post-exercise hyperglycemia was temporary; therefore, treating it with insulin was not recommended [43]. However, a more recent study recommended using 100% to 150% correction based on a patient’s usual insulin correction factor to prevent post-HIIT hyperglycemia [44].

Haymond et al. evaluated a new approach to prevent post-exercise hypoglycemia that involved giving a small dose of glucagon before exercise [45]. They demonstrated that administering 150 µg of subcutaneous glucagon was as effective as oral glucose tablets in managing mild to moderate hypoglycemia. This approach prevents unnecessary excess caloric intake associated with oral glucose tablet ingestion [46]. Rickels et al. showed that mini-dose glucagon (150 µg subcutaneous) given before exercise may be more effective than insulin reduction in preventing exercise-induced hypoglycemia [47]. When compared to carbohydrate ingestion, administering low-dose glucagon before exercise may result in less post-exercise hypoglycemia [47].

Carbohydrate management

The amount of carbohydrates athletes should consume depends on the level of exercise they undertake. For low-intensity training, the carbohydrate recommendation is 3 to 5 g/kg/d, and for moderate to high-intensity exercise of four to five hours/day, the carbohydrate recommendation is 10 to 12 g/kg/d [48].

A simple algorithm to remember is to consider ingestion of 15 to 30 g of carbohydrates for each 30 minutes of exercise. This can be done in lieu of or in addition to decreasing the insulin dose [49].

Carbohydrate loading before events can be used to postpone fatigue and extend the duration of steady-state exercise by about 20% and workload by 2% to 3% [50]. Many strategies exist that depend on the level of intensity of exercise the athlete will undertake. Athletes may ingest a high-carbohydrate, high-glycemic-index diet for one day before an event, with one day of inactivity, or for three days before an event with a two-day taper of exercise and one day of inactivity [51]

Blood glucose monitoring

As mentioned above, the blood glucose response to exercise is variable and depends on several factors [7]. Frequent monitoring of blood glucose before, during, and after exercise is crucial to prevent serious adverse events and to ensure the best performance. In the hour preceding exercise, blood glucose checks every 30 minutes are recommended to plan for insulin adjustments or for extra carbohydrate intake before exercise. During exercise, frequent glucose monitoring should continue at the same rate, once every 30 minutes, to understand the blood glucose direction and to anticipate hyper or hypoglycemia [12]. Post-exercise, glucose monitoring every two hours for up to four hours is recommended to prevent post-exercise hypoglycemia [52-53].

A continuous glucose monitor (CGM) is a device that uses a small flexible metal wire (sensor) inserted just below the skin where it generates a small electric signal in response to interstitial glucose. This signal is converted into a blood glucose reading and transmitted wirelessly at certain intervals to a dedicated receiver for display to a user. Previous studies have shown that CGMs have reasonable accuracy during aerobic and anaerobic exercise, including HIIT [54-55]. The use of a CGM has also been helpful in detecting post-exercise hypoglycemia and aiding patients with T1DM in managing their blood glucose [56-57]. It is important to note that during episodes of hypoglycemia, the drop in CGM glucose lags behind the drop in blood glucose. Therefore, when suspecting hypoglycemia during exercise, patients should confirm it with a capillary glucose measurement [58-59].

Detection and treatment of hypoglycemia

Even with our best efforts, hypoglycemia does happen and should be recognized and treated. Symptoms may vary among individuals and may include autonomic symptoms like palpitations, tremors, restlessness, and anxiety; these symptoms are catecholamine-mediated. Other symptoms of hypoglycemia include sweating, hunger, and paresthesia, which are acetylcholine-mediated. Some may develop neuroglycopenic symptoms, including dizziness, weakness, confusion, erratic behavior, or even seizures and loss of consciousness [60].

The “Rule of 15” can be followed for treatment [61]. If the blood sugar level is 70 mg/dL or lower, the athlete should be given 15 g of carbohydrates and tested again after 15 minutes. The process is repeated until the blood sugar is above 70 mg/dL [62]. Practical choices for carbohydrate sources in the field are glucose tablets (4 tablets = 16 g of carbohydrates) and fruit juice (4 oz = 15 g of carbohydrates).

If an athlete with hypoglycemia is not awake enough to take a carbohydrate source orally, glucagon should be injected intramuscularly [63]. Ideally, the athlete should have access to glucagon at all times. Athletes with T1DM are advised to make people close to them aware of where they keep their glucagon kit and how it is used. In the field, the coach, a family member, or a trusted teammate might have to administer glucagon to the athlete. Care should be taken to ensure that the glucagon kits are not expired.

## Conclusions

DM is the most common metabolic disease, and T1DM is the type more likely to be managed in sports and athletics, likely related to the younger age of patients with T1DM. Hypoglycemia, the most common acute adverse event during and after exercise, can negatively impact an athlete’s performance and health and should be avoided. There are several recommendations on how to prevent hypoglycemia during and after exercise. The authors advise athletes and care providers to choose and apply a recommended approach as a starting point, with the goal of individualizing management for each athlete by continuous monitoring and adjustment. The use of a CGM is becoming more prevalent in preventing hypoglycemia in general and has become very useful for monitoring sugar levels during and after exercise. The US Food and Drug Administration has approved the use of data from one CGM system to make DM treatment decisions.
